# Developing a provisional, international Minimal Dataset for Juvenile Dermatomyositis: for use in clinical practice to inform research

**DOI:** 10.1186/1546-0096-12-31

**Published:** 2014-07-21

**Authors:** Liza J McCann, Katie Arnold, Clarissa A Pilkington, Adam M Huber, Angelo Ravelli, Laura Beard, Michael W Beresford, Lucy R Wedderburn

**Affiliations:** 1 Alder Hey Children’s NHS Foundation Trust, Eaton Road, Liverpool L12 2AP, UK; 2 Rheumatology Unit, UCL Institute of Child Health, University College London, London, UK; 3 Centre for Adolescent Rheumatology at University College London, University College London Hospital, London, UK; 4 Great Ormond Street Hospital, London, UK; 5 IWK Health Centre and Dalhousie University, 5850 University Avenue, Halifax, NS B3K 6R8, Canada; 6 Università degli Studi di Genova and Istituto Giannina Gaslini, Largo G. Gaslini 5, 16147 Genoa, Italy; 7 Department of Women’s and Children’s Health, Institute of Translational Medicine, University of Liverpool, Liverpool, UK

**Keywords:** Juvenile dermatomyositis, Idiopathic Inflammatory myopathy, International, Collaboration, Dataset, Core set, Disease activity

## Abstract

**Background:**

Juvenile dermatomyositis (JDM) is a rare but severe autoimmune inflammatory myositis of childhood. International collaboration is essential in order to undertake clinical trials, understand the disease and improve long-term outcome. The aim of this study was to propose from existing collaborative initiatives a preliminary minimal dataset for JDM. This will form the basis of the future development of an international consensus-approved minimum core dataset to be used both in clinical care and inform research, allowing integration of data between centres.

**Methods:**

A working group of internationally-representative JDM experts was formed to develop a provisional minimal dataset. Clinical and laboratory variables contained within current national and international collaborative databases of patients with idiopathic inflammatory myopathies were scrutinised. Judgements were informed by published literature and a more detailed analysis of the Juvenile Dermatomyositis Cohort Biomarker Study and Repository, UK and Ireland.

**Results:**

A provisional minimal JDM dataset has been produced, with an associated glossary of definitions. The provisional minimal dataset will request information at time of patient diagnosis and during on-going prospective follow up. At time of patient diagnosis, information will be requested on patient demographics, diagnostic criteria and treatments given prior to diagnosis. During on-going prospective follow-up, variables will include the presence of active muscle or skin disease, major organ involvement or constitutional symptoms, investigations, treatment, physician global assessments and patient reported outcome measures.

**Conclusions:**

An internationally agreed minimal dataset has the potential to significantly enhance collaboration, allow effective communication between groups, provide a minimal standard of care and enable analysis of the largest possible number of JDM patients to provide a greater understanding of this disease. This preliminary dataset can now be developed into a consensus-approved minimum core dataset and tested in a wider setting with the aim of achieving international agreement.

## Background

Juvenile dermatomyositis (JDM) is a severe, autoimmune, inflammatory myositis that can cause death and major long-term health problems [1-3]. There are major gaps in knowledge regarding epidemiology, pathogenesis, response to medication and long-term outcome of the disease [4]. Although most children with JDM now survive to adulthood (5 year survival > 95%) [5], a significant number (70-80%) have on-going disease activity [1-3]. JDM differs in many ways from myositis in adults [5]. As JDM is rare, affecting 2–3 per million children per year [6,7], international collaboration between paediatric and adult JDM research groups is essential to undertake clinical trials, understand the disease, and improve long-term outcome. The first prospective randomised trial in myositis (evaluating rituximab in refractory disease) enrolling both paediatric and adult patients through international collaboration was recently published [8]. A collaborative randomised trial of prednisolone, methotrexate and ciclosporin in JDM has recently been completed [9]. Aside from these, there remains a significant paucity of robust evidence regarding therapeutic interventions for JDM in the paediatric and adolescent age group.

To date, two parallel processes have led to the development of two ‘core sets’ for JDM in America (via the International Myositis and Clinical Studies [IMACS] group) [10-12] and in Europe (via Paediatric Rheumatology International Trials Organisation, PRINTO) [13-15]. These core sets overlap but differ in important details and lack international agreement. They were developed with the main purpose for clinical trials and therefore can be difficult to use in routine clinical practice due to time constraints.

Disease specific registries, particularly when linked to a biobank of biological specimens, can be powerful tools enabling epidemiological research, identification of risk factors to develop disease (such as gene-environment interaction) and identification of patients for clinical trials [16]. They can also drive a minimal standard of care by encouraging capture of important variables for disease monitoring within clinical use. In addition, registries have the potential of providing a platform for the development of robust pharmacosurveillance and post-marketing drug safety monitoring, exemplified by the Childhood Arthritis and Rheumatology Research Alliance (CARRA) Consolidated Safety Registry (CoRe) [17]. The Juvenile Dermatomyositis Cohort Biomarker Study and Repository, UK and Ireland (JDCBS) provides a large prospective collection of juvenile idiopathic inflammatory myopathies (currently >430 patients) with a repository of linked biological and serological specimens [18]. In 2010, CARRA initiated a multi-centre observational registry in the United States to create a clinical database for the major rheumatic diseases, [19] which has reported data from 384 JDM patients enrolled within the first 2 years [20]. In 2008, a European initiative was taken to develop a web-based registry for adult myositis patients, Euromyositis [16], which is currently being expanded to include JDM patients through collaboration with the UK Juvenile Dermatomyositis Research Group (JDRG) [18].

The concept underpinning disease-specific registries is a user-friendly, accessible database that can be useful in clinical practice and encourages data-entry for research purposes. In order to achieve this, databases need to collect important information that helps to define disease outcome and if possible inform treatment, but not be so extensive that it is too time-consuming. Ideally, it should be linked to the patient record to avoid duplication [16] and yet be protected so that patients are not identifiable within a secure web-based system when data are extracted for research purposes.

Currently, existing databases within JDCBS, CARRA and Euromyositis have dataset parameters that are partially overlapping but use different data items. Analysis of information held within these individual data collections has led to a greater understanding of the disease, resulting in numerous publications on JDM [18,20]. However, many important questions remain unanswered and depend on analysis of larger numbers of patients than are currently available in individual collections. To address this, this present study aimed to scrutinize the existing collaborative datasets to propose a common minimal core data set comprising clinical, laboratory and patient/parent reported measures for patients with JDM. We aimed for such a core data set to be clinically practical, incorporate key variables that allow accurate assessment of disease activity and measure change (such as response to treatment), and for the data set to be used in the development of future clinical trials in JDM. Such a common dataset, could form the basis for a future consensus-driven, internationally approved minimum core dataset that can be rapidly incorporated into national and international collaborative efforts, including existing prospective databases, and be available for use in randomised controlled trials and for treatment / protocol comparisons in cohort studies.

## Methods

A group of five JDM experts from the UK, Italy and Canada (LM,CP,LW,AH,AR) representative of the major groups studying JDM and maintaining databases (CARRA, UK JDCBS, PRINTO) formed a working group which aimed to produce a provisional minimal dataset for JDM [21].

A literature search, carried out by one author (LM), aimed to identify any variables potentially missing from current database collections but used within validated research tools. The search (EMBASE, MEDLINE, September 2012; main search terms ‘dermatomyositis’ OR ‘myositis’ AND ‘juvenile’ OR ‘childhood’ AND ‘database’ or ‘dataset’ or ‘consensus’ or ‘core set) identified 272 publications. Articles containing variables used within published ‘core sets’ for research purposes (PRINTO/IMACS) and variables used within articles describing disease classification, diagnostic criteria, clinical characteristics, disease monitoring, outcome assessment/response to treatment or definition of inactive disease were listed for initial consideration and presented to the group.

A detailed analysis of variables from three existing large registries of patients with Idiopathic Inflammatory Myopathies (IIM), namely JDCBS (http://www.juveniledermatomyositis.org.uk), CARRA (http://www.carragroup.org), and Euromyositis (http://www.euromyositis.eu), as well as those used in a Paediatric Rheumatology INternational Trials Organisation (PRINTO) coordinated retrospective study analyzing 490 JDM patients [1], was undertaken. Each variable common to at least two databases was listed and scrutinized for usefulness within its clinical context and for research studies by e-mail and teleconference discussion within the expert group [21]. Variables were only included in the provisional dataset when 100% of group members agreed that they were potentially important for clinical use and/or future research.

To inform discussion, a separate detailed analysis of the UK JDCBS database was carried out in July 2011 to determine the number of times that variables were completed accurately. The JDCBS had ethical approval from the North Yorkshire Multi-Centre Research Ethics Committee and this study was also approved by the Steering Committee of the UK JDM Cohort Study. At this time the JDCBS had recruited 285 children with sufficient data available for analysis on 275 cases [18] of which 175 (63.3%) had been recruited prospectively within 12 months of onset. Any variable completed <50% of the time was scrutinized and discussed within the expert group to determine importance of inclusion versus reliability of completion and to see if questions could be asked in a more steadfast way to encourage completion.

Definitions of variables used by the individual databases were reviewed. Those used within the Euromyositis database were modified for paediatric use. A combination of these definitions, with the aid of agreed and published descriptions of cutaneous manifestations of IIM [22], were used to form a glossary/definition of variables.

## Results

Analysis of the four datasets (JDCBS, CARRA, Euromyositis, PRINTO) showed that 19 variables were common to all four datasets, 16 were common to three datasets, and 24 were common to two datasets (Table 1). Although only one database (JDCBS) included data on the use of physiotherapy, this variable was retained within the treatment listing as it was felt to be important by all JDM experts. Likewise, malignancy was retained as a variable, despite being recorded by Euromyositis alone. Therefore a total of 61 variables were scrutinized for potential inclusion. An additional 29 variables were identified as only being in one of the four datasets and excluded from further consideration at this stage. Cross-referencing of variables listed from the literature search and included within research tools such as the Myositis Disease Activity Assessment (MDAA) and Disease Activity Score (DAS) [15] was carried out to ensure that members of the working groups were satisfied that all variables important for clinical use were included. This process informed discussion but did not lead to the inclusion of any additional variables.

**Table 1:** Variables common to more than two datasets, considered for inclusion in provisional minimal dataset

**Variables common to four datasets (n = 19)**	**Variables common to three datasets (n = 16)**	**Variables common to two datasets (n = 24)**
Date of birth and patient code	Patient name	Postcode
Gender	Patient centre	Death
Ethnicity	Diagnosis date	Fatigue due to JDM
Family history	JDM rash	Distribution of rash
Onset date	Skin erythema	Abdominal pain due to JDM
Height/Weight	MRI consistent with JDM at diagnosis	Neurological involvement
Symmetrical muscle weakness	CMAS	Weight loss due to JDM
Gottron’s or heliotrope rash	Gottron’s sign	Alopecia due to JDM
Skin ulcers	Shawl sign	Nail-fold changes
Dysphagia or dysphonia	Gastro-intestinal disease / ulceration due to JDM	Dysphagia
Calcinosis	Fever due to JDM	Dysphonia
Cardiac involvement	Abnormal pulmonary function tests	Respiratory symptoms
Arthritis	Contractures	Myalgia
Interstitial lung disease	Lipodystrophy	Eye disease (glaucoma/cataract)
MMT8	Autoantibody data (more than ANA)	Raynaud’s
Muscle enzymes elevated	Physician global assessment	Parental Visual Analogue Scale (VAS)
EMG changes consistent with JDM at diagnosis		Pain VAS
Muscle biopsy consistent with JDM		CHAQ
Medication history		MITAX/MYOACT
		Hospitalisation since last visit
		School absence
		Other significant diagnosis
		Sporting activities
		Pubertal delay

All registries or data collections included patient demographics, date of onset of symptoms, date of diagnosis and growth indicators. They all included diagnostic indicators of JDM, including evidence of a characteristic skin rash, proximal muscle weakness, muscle enzyme elevation, electromyogram (EMG) and muscle biopsy evidence of myositis. There were some differences in how these data were documented, particularly when comparing adult and paediatric registries. All databases asked about the presence of skin ulceration, calcinosis, arthritis and cardiac/respiratory involvement although there were differences in how the questions were asked and data presented. All databases asked for medication history.

Two databases asked about neurological involvement. Capture of systemic symptoms (weight loss, fatigue, myalgia, irritability) varied between databases. There were differences in how disease activity over time was monitored, although the majority of databases included patient/parent Visual Analogue Scale (VAS), a Health Assessment Questionnaire (CHAQ/HAQ), physician global VAS, and a measure of muscle strength; either the Childhood Myositis Assessment Scale (CMAS) and/or Manual Muscle Testing (MMT) [23]. The PRINTO study used the Disease Activity Score (DAS) whereas Euromyositis included the Myositis Intention to Treat Activity Index (MITAX) and the Myositis Disease Activity Assessment Visual Analogue Scales (MYOACT) [23].

The presence of Raynaud’s phenomenon was retained due to the potential significance of progression of childhood onset myositis into scleroderma or myositis/scleroderma overlap in adult life [24]. The presence of mechanics hands and malignancy were each retained despite being infrequent in paediatrics, in order to compare with adult datasets.

Scrutiny of the UK JDCBS database, which included routine collection of 54 out of a total of 61 possible variables in the provisional minimal database, showed that at baseline, 8/54 (15%) variables were completed in <50% of patients. Specific concerns were noted with completion of family history, Childhood Health Assessment Questionnaire, Child Health Questionnaire, parental visual analogue scale of disease activity, pain visual analogue score, as well as nail-fold capillary changes, lipodystrophy and Raynaud’s phenomenon. Analysis of completion rates for two of these variables (nail-fold changes and Raynaud’s) could have been confounded by the fact that they were not included in the original data collection forms, but added at a later date. With prospective collection, parameters performing poorly (completed in <50% patients) were MRI, muscle biopsy, antibody and pulmonary function test results. Discussion within the expert group confirmed the importance of these parameters but led to rephrasing of questions to support future enhanced data completion. For example, the question ‘pulmonary involvement due to myositis’ was favoured as opposed to asking clinicians to document the results of pulmonary function tests.

**Figure 1:**
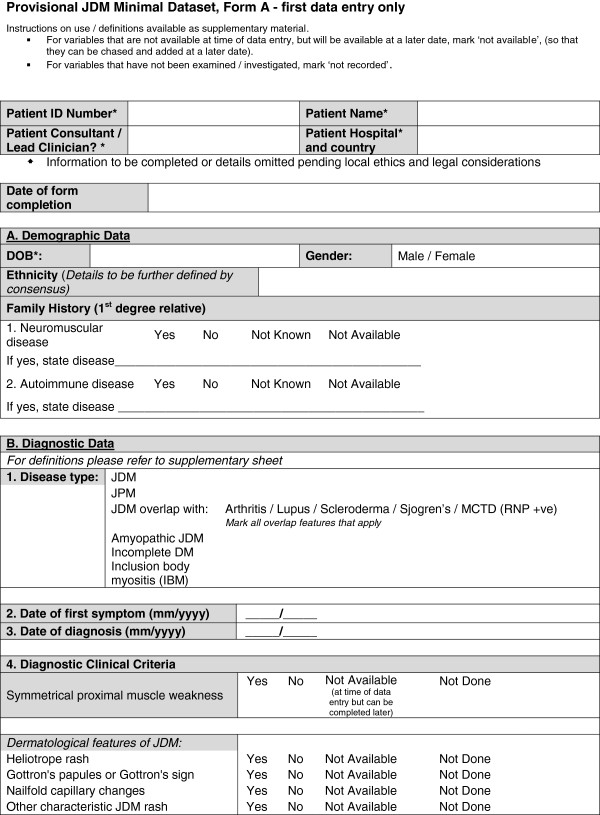
Provisional JDM minimal dataset Form A (first data entry only), page 1 –demographic and diagnostic data.

**Figure 2:**
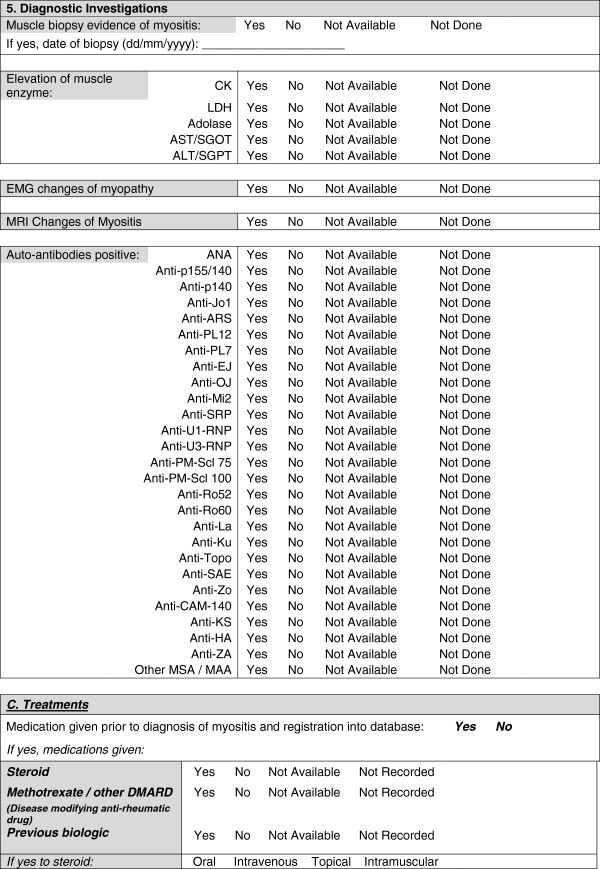
Provisional JDM minimal dataset Form A, (first data entry only), page 2 – diagnostic investigations and treatments.

**Figure 3:**
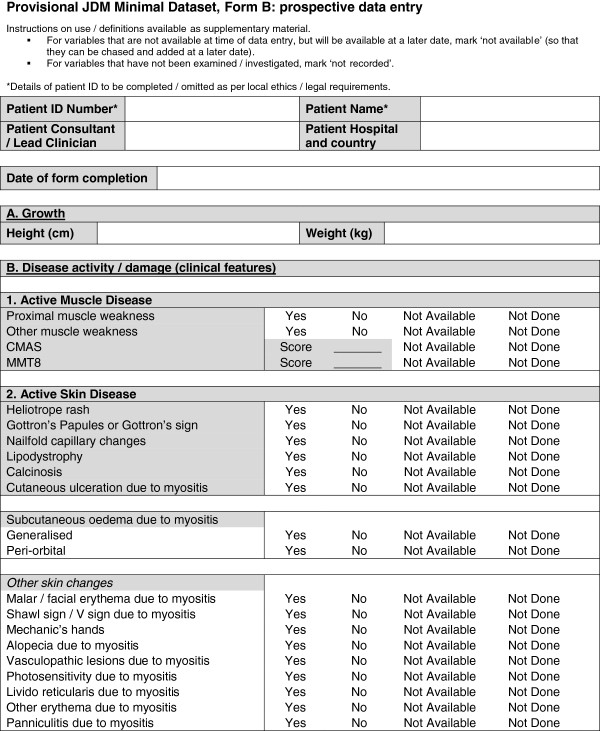
Provisional JDM minimal dataset, Form B, (prospective data collection), page 1 – disease activity and damage.

**Figure 4:**
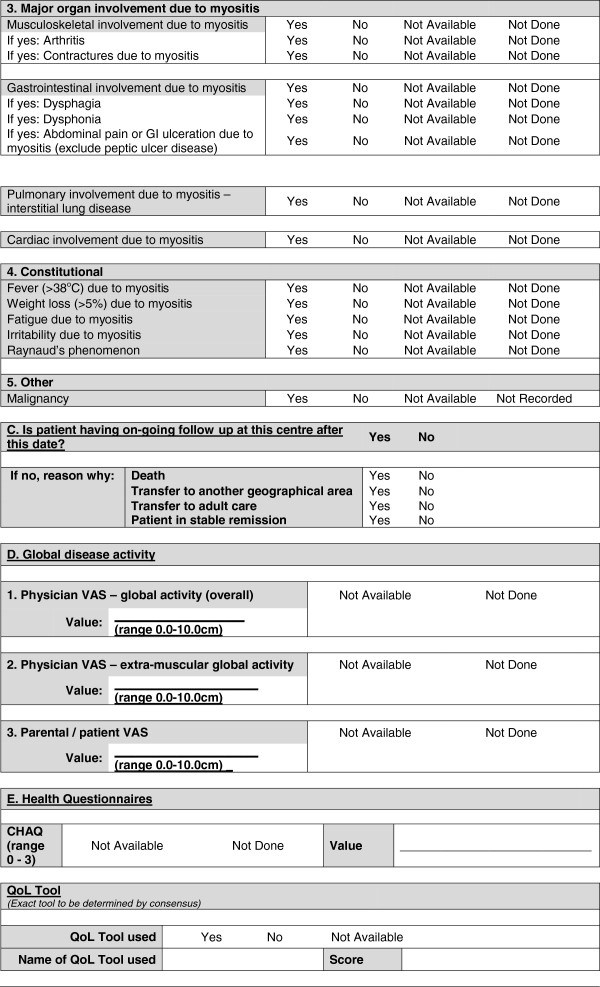
Provisional JDM minimal dataset, Form B, (prospective data collection), page 2 –disease activity/damage (continued).

**Figure 5:**
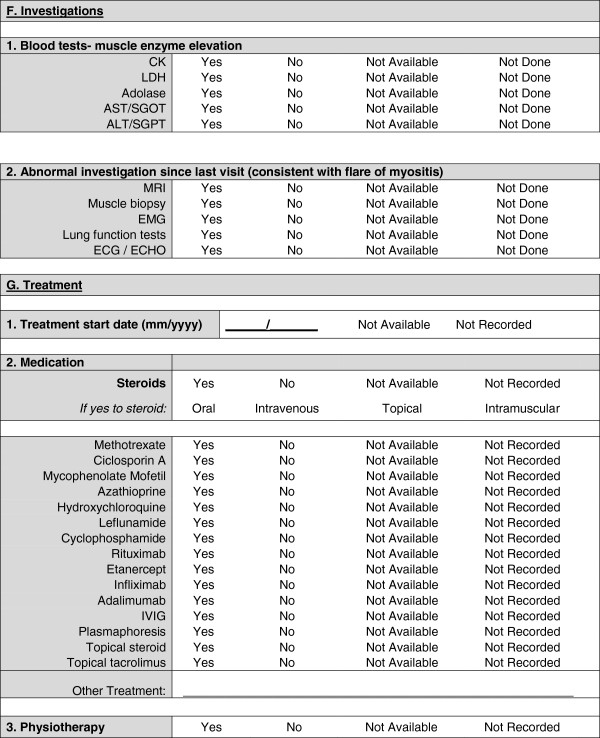
Provisional JDM minimal dataset, Form B, (prospective data collection), page 3 – investigations and treatment.

A provisional minimal dataset was therefore established from the existing collaborative datasets for use in clinics and inform clinical trials and research studies. This provisional dataset was then configured into user-friendly forms (Figures 1, 2, 3, 4 and 5) and organized into ten domains. Three of these domains (demographic data, diagnostic data and treatment prior to diagnosis) are questions that would be asked at first presentation only (Figures 1 and 2). The remaining seven domains (growth, clinical features, ongoing follow up, physician reported outcome measures, patient/parent reported outcome measures, investigations, treatment) are relevant for every patient visit (Figures 3, 4 and 5). A glossary for form A (Figures 1 and 2) and form B (Figures 3, 4 and 5) are available as Additional file 1 (Form A: provisional definitions, Form B: provisional definitions).

Our working group includes a member of the Euromyositis Steering Committee and in collaboration with the Euromyositis Research Group, we have used the provisional dataset as a platform to create a paediatric-specific webpage for the Euromyositis database, ensuring compatibility with the web-based system used to collect data on adult-onset idiopathic inflammatory (http://www.euromyositis.eu).

Further work will develop this preliminary dataset into a consensus-approved minimum core dataset, tested in a wider setting with the aim of achieving international agreement.

## Discussion

International collaboration among myositis experts within IMACS and PRINTO has led to the development of two core set measures for JDM [10-15]. These are important tools predominantly designed for research and clinical trials. Time constraints limit the use of these in routine clinical practice. However, the tools capture important variables that measure disease activity and damage in JDM. In this study we have attempted to capture these individual variables within a provisional minimal dataset with the aim of producing a platform for recording important information within routine clinical practice, and providing a minimum standard of care for JDM. This will have the added benefit of being able to be used to inform future research and will therefore compliment the existing IMACS/PRINTO core sets.

Our group has scrutinized variables collected within four existing databases, in addition to variables used within established research tools and published literature, to form a provisional minimal dataset for JDM. Further development is now needed through a formal consensus-driven process with the aim of ultimately achieving a unified internationally agreed minimal dataset for JDM for clinical use that can also inform research.

The development of an internationally agreed minimal dataset could have numerous potential benefits. Unification of data collection internationally will significantly enhance collaborative efforts and communication between groups (whilst respecting data protection/ownership) and allow analysis to be undertaken on the largest possible number of JDM patients. This has the potential to improve understanding of disease phenotype and outcome in a way that will define research questions and underpin prioritisation of future clinical trials. Analysis of a large number of JDM patients with international collaboration has the potential to help answer fundamental questions including characteristics of patients who develop JDM, how patients develop the disease, time to diagnosis (and raise awareness leading to quicker diagnosis), which tests are most useful for diagnosis and which features predict a more severe disease course. Increasingly, distinct clinical subtypes are recognised within IIM, associated with myositis specific antibodies, but differences are seen in adults and children [25]. Our understanding of the mechanisms that underlie these differences is limited by lack of studies directly comparing adults and children in sufficient numbers. A minimal dataset including disease characteristics and antibody profiling may help to answer these questions with the potential of directing subsequent clinical trials of treatment strategies on disease stratified by serology. It is increasingly recognized that patient reported perspectives reveal disease aspects not covered by traditional outcomes in myositis [26]. A minimal dataset will include patient/parent reported outcome measures and work is ongoing to develop new JDM-specific patient/reported outcome measures (the Juvenile Dermatomyositis Multidimensional Assessment Report, JDMAR) [27]. With international collaboration, patient reported outcome measures, such as this, could be evaluated in large numbers of patients, helping to predict effectiveness for their use in future clinical trials.

A large international collection has the potential to allow detailed evaluation of ‘real world’ treatment interventions used in clinical practice – based on clinician’s intention to treat, to see if one treatment is better than another. The CARRA database includes the option of clinicians choosing which of a predefined number of treatment strategies to use within the context of a consensus treatment plan [28]. If other studies contain the same minimal dataset, outcome of patients on different treatments can be compared and evaluated.

International collaboration has the potential to enable comparative studies of differences between adult and juvenile dermatomyositis. Our group has collaborated with Euromyositis to produce a paediatric specific web page for data entry for patients with JDM (http://www.euromyositis.eu). This web-page complements current data collection on adult myositis and will allow comparison between the two groups. New variables that are proposed as part of this international minimal dataset could be incorporated into the Euromyositis data collection. An important part of this collaboration is to ensure that children entered into adult networks have paediatric-specific outcomes (such as growth) recorded. Collaborative efforts in JDM have led to data-driven approaches such as recently published PRINTO-driven criteria for defining clinically inactive disease in JDM [29]. A minimal dataset will allow validation of such approaches in large cohorts of patients across several prospective data collections.

A minimal dataset will provide a framework within which all children with JDM can be monitored in a similar way, including important variables, as a minimum standard of care. The dataset will prompt clinicians to check important clinical features including muscle strength, skin activity and investigations each time a child with JDM is seen. This is important since JDM is a rare disease and some clinicians are not seeing large numbers of patients with the disease. The importance of defining minimal standards of care has been recognized internationally and members of our group are involved in a European effort to produce standards of care within a Single Hub and Access Point for Paediatric Rheumatology in Europe (SHARE) for JDM as well as other paediatric rheumatological conditions [30].

A provisional minimal dataset has been defined by consensus between a group of experts in JDM, representative of the major national and international JDM collaborative efforts. This now needs to be refined and tested for its usefulness and acceptability in a wider international setting. In order to achieve this, a formal consensus-driven methodology is required of all stakeholders. This will include a Delphi process to gain widespread opinion via the established JDM networks that will help to inform a more detailed face-to-face nominal group consensus process by internationally representative JDM experts.

## Conclusion

JDM is a rare but serious inflammatory condition with an appreciable risk of long-term morbidity. There are many unanswered questions around epidemiology, pathogenesis, treatment and outcome of the disease. The provisional dataset developed to date will be used as a template to aid a formal multi-stage consensus process, involving large groups of clinicians caring for children with JDM, with the aim of developing a feasible, internationally agreed minimal dataset for clinical use that can inform research.

## Abbreviations

JDM: Juvenile dermatomyositis; IIM: Idiopathic Inflammatory Myopathy; JDRG: Juvenile Dermatomyositis Research Group (UK and Ireland); JDCBS: Juvenile Dermatomyositis Cohort Biomarker Study and Repository, UK and Ireland; CARRA: Childhood Arthritis and Rheumatology Research Alliance; PRINTO: Paediatric Rheumatology INternational Trials Organisation; IMACS: International Myositis and Clinical Studies group; OMERACT: Outcome Measures in Rheumatology; EMG: Electromyogram; MRI: Magnetic Resonance Imaging; CHAQ: Childhood Health Assessment Questionnaire; VAS: Visual Analogue Scale; CMAS: Childhood Myositis Assessment Scale (CMAS); MMT: Manual Muscle Testing (MMT); DAS: Disease Activity Score (DAS); MITAX: Myositis Intention to Treat Activity Index (MITAX); MYOACT: Myositis Disease Activity Assessment Visual Analogue Scales.

## Competing interests

The authors declare that they have no competing interests.

## Authors’ contributions

LM, CP, LW, AH, AR involved in all stages of the development of minimal dataset. LB was involved in scrutinising the UK JDCBS (carrying out data queries) and administrative tasks. KA was involved in scrutinising the UK JDCBS database, carrying out administrative tasks and developing the database format / clinical forms. LM, CP, MB, LW involved in intellectual content and obtaining funding to take this work forward. All authors read and approved the final manuscript.

## Supplementary Material

Additional file 1 **Form A: provisional definitions, Form B: provisional definitions (to be refined further as part of on-going work)**[31-37] Click here for file
