# Supplementary material for: Developing a provisional, international Minimal Dataset for Juvenile Dermatomyositis: for use in clinical practice to inform research
Source: Pediatr Rheumatol Online J. 2014 Jul 21;12:31. doi: 10.1186/1546-0096-12-31 (PMC4113599; doi:10.1186/1546-0096-12-31)
Supplement: Additional file 1 — Form A: provisional definitions, Form B: provisional definitions (to be refined further as part of on-going work)[31-37] [file 1546-0096-12-31-S1.zip › add1/8866834211209620_add1.doc]

**Form A: Provisional definitions**

For each variable mark yes (present), no (not present), not available (not available at time of entry but can be chased and added at a later date) or not done (not examined or investigated, hence not available at a later date).

**Patient Information**

**Patient ID Number:** Country specific person identification number or social security number. This field will have different use in different countries based on local legislation. You may decide to use hospital specific project numbers in this field.

**Patient name and patient birthday** are the core identifiers in the system, when you later look for the patient. If your local legislation does not allow this, the item can be omitted. Data entry also allows entry of year and month of birth without day of birth.

**Consultant:** Typically if the 'typing' user is not the consultant

**Patient Hospital** Used when patient followed at external hospital or clinic - other than the 'typing' hospital

**A. Demographic Data**

**Ethnicity**- select mixed race by selecting more than one ethnicity. Ethnicity categories to be further defined by consensus during on-going work.

**Family History**

i) Neuromuscular diseases, such as: Non-inflammatory inclusion body myositis, muscular dystrophy, metabolic myopathy, mitochondrial myopathy, drug or toxin associated myopathy, infectious myopathy, endocrine myopathy, motor neurone disease or other (specify).

ii) Autoimmune disease, such as: autoimmune thyroid disease, Type 1 (insulin dependent) diabetes, Rheumatoid arthritis or Juvenile Idiopathic Arthritis, Systemic Lupus Erythematosis, seronegative spondyloarthropathies, Multiple Sclerosis, inflammatory bowel disease (Crohn’s / Ulcerative Colitis), Psoriasis, Sjogren’s, Scleroderma, Addison’s disease, Pernicious anaemia, Myasthenia Gravis, Primary Biliary Cirrhosis, mixed connective tissue disease, vitiligo, autoimmune haemolytic anaemia, coeliac disease, pemphigus, alopecia areata, Behcet’s, myositis (dermatomyositis / polymyositis), Goodpasture’s, Vasculitis (polyangiitis with granulomatosis, polyarteritis nodosa, Kawasaki, Henoch Schonlein Purpura) or other (specify).

**B. Diagnostic Data**

**Diagnosis Type**

i) Juvenile Dermatomyositis
ii) Juvenile Polymyositis

Classification criteria for polymyositis and dermatomyositis [31]:

1. Symmetrical proximal muscle weakness.

2. Elevation of the serum enzymes, especially creatine phosphokinase, aldolase, aspartate aminotransferase, alanine aminotransferase and lactate dehydrogenase.

3. Abnormal electromyogram with myopathic motor unit potentials, fibrillations, positive sharp waves, increased insertional irritability and spontaneous bizarre high frequency discharges.

4. Muscle biopsy evidence of necrosis, phagocytosis, regeneration, degeneration, perifascicular atrophy, and an interstitial inflammatory infiltrate.

5. Typical skin rash of DM (see below).

Definite dermatomyositis: criterion 5 and three of criteria 1-4, probable dermatomyositis: criterion 5 and two of criteria 1-4, possible dermatomyositis criterion 5 and one of criteria 1-4.

Definite polymyositis: criteria 1-4, probable polymyositis three of criteria 1-4, possible polymyositis: two of criteria 1-4.

• Symmetry is intended to denote bilateral but not necessary equal involvement.

• The application of these criteria assumes that known infectious, toxic, metabolic, dystrophic, or endocrine myopathies have been excluded by appropriate evaluations.

Exclusions:

• Evidence of central or peripheral neurologic disease, including motor-neuron disorders with fasciculations or long-tract signs, sensory changes, decreased nerve conduction times, and fibre-type atrophy and grouping on muscle biopsy.

• Muscle weakness with a slowly progressive, unremitting course and positive family history or calf enlargement to suggest muscular dystrophy.

• Biopsy evidence of granulomatous myositis such as with sarcoidosis.

• Infections, including viral (influenza, coxsackie B, adeno, parainfluenza, parvovirus, HIV, hepatitis B), bacterial (staphylococcus, streptococcus, toxoplasmosis, Lyme borreliosis, Yersinia, Legionella pneumphilia), fungal (sporotripchosis, histoplasmosis, mucormycosis, Cryptococcus, candidiasis) or parasitic (protozoa, cestodes, nematodes).

• Endocrinopathies such as hypothyroidism, hyperthyroidism, hyperparathyroidism, hypoparathyroidism, diabetes mellitus, Cushing’s syndrome, Conn’s syndrome, acromegaly.

• Myasthenia gravis.

• Recent use of various drugs and toxins, such as glucocorticoid, hydroxychloroquine, diuretics, amphotericin B, cimetidine, growth hormone, vincristine, statins, interferon-alpha, clofibrate and alcohol.

• Metabolic abnormalities: excess / deficiency of potassium, calcium, phosphorus, magnesium, or vitamin D.

• Rhabdomyolysis as manifested by gross myoglobinuria related to strenuous exercise, infections, crush injuries, occlusion of major limb arteries, prolonged coma or convulsions, high voltage accidents, heat stroke, the malignant-hyperpyrexia syndrome, and envenomation by certain sea snakes.

• Metabolic disorders such as muscle glycogenoses (glycogen storage disorders), lipid-storage disorders, mitochondrial myopathies.

• Other rheumatic conditions: juvenile arthritis, systemic lupus erythematosis, scleroderma (unless as part of an overlap syndrome with myositis / mixed connective tissue disorder) or idiopathic vasculitis.

For more information on differential diagnosis, refer to Washington Neuromuscular Disease Centre [32].

iii). Overlap myositis - myositis plus another defined connective tissue or autoimmune disease (to be defined further after consensus as part of on-going work).

iv). Amyopathic dermatomyositis (ADM) – rare in children.

ADM is defined as a rare group of the IIM, in patients who have classic cutaneous findings confirmed by skin biopsy, in the absence of clinical, laboratory, or histologic evidence of myositis after at least 2 years of follow up [33].

Hallmark cutaneous findings of dermatomyositis

Pathognomonic

• Gottron’s papules: erythematous to violaceous papules over the extensor surfaces of the metacophalangeal joints and interphalangeal joints, knees, ankles, elbows.

Highly characteristic

• Heliotrope rash with or without oedema: violaceous to erythematous discrete or confluent macules over the upper eyelids.

Characteristic

• Symmetric violaceous/erythematous or hyperpigmented patches.

• Gottron’s sign (symmetrical erythematous macules or patches in the same distribution as Gottron’s papules).

• Shawl sign (erythema over the upper back, posterior neck and shoulders, sometimes extended to lateral arms)

• V sign (erythema over lower anterior neck and upper anterior chest).

• Holster sign (erythema confined to the lateral thigh / hip).

• Linear extensor erythema (confluent macular erythema of skin overlying the extensor tendon sheaths).

• Facial erythema / malar rash (usually sparing naso-labial folds).

• Mechanic's hands (unusual in children): hyperkeratotic lesions on the palmar and lateral aspects of the digits).

• Periungual erythema (erythema of the skin and dilatation of the blood vessels bordering the base and lateral aspects of the nail plate), nail-fold telangiectasias (capillary loop changes including dilatation, tortuosity, vessel drop out) and cuticular overgrowth (enlargement and overgrowth of the cuticle on the nail plate – more common in adult DM).

• Poikiloderma vasculare atrophicans (hyperpigmented or hypopigmented macules interspersed with fine telangiectasia and cutaneous atrophy).

• Photosensitivity (sun exposed erythema).

• Pruritus.

Cutaneous findings more common in juvenile dermatomyositis

• Vasculopathic lesions (including livido reticularis, ulceration, gingival erythema, periungual erythema / telangiectasia and digital infarction.

• Cutaneous calcinosis (dystrophic calcification involving the skin, subcutaneous tissue, fascia or muscle).

• Lipodystrophy (loss of subcutaneous fat) – may be localised, partial or generalised.

Rare or uncommon findings in dermatomyositis

• Erythroderma (extensive areas of confluent erythema).

• Anasarca (extreme generalised oedema).

• Vesiculobullous lesions.

• Acquired icthyosis.

• Generalized and infrapatellar hypertrichosis.

• Follicular papules and hyperkeratosis (Wong type dermatomyositis).

• Non-scarring alopecia.

• Leukocytoclastic vasculitis (small-vessel or necrotizing vasculitis).

• Livedo reticularis.

• Panniculitis.

• Mucinous papules and plaques.

• Flagellate erythema.

For more information, refer to Dugan, EM et al [22].

v). Incomplete myositis

Incomplete myositis is entered when you suspect myositis, but case does not fulfill diagnostic criteria for classical DM, PM, JDM, amyopathic DM or IBM. Reasons for not having the concrete diagnosis could be multiple, such as inability to access all necessary investigations, fulfillment of only some criteria with a strong suspicion for myositis, presence of only complete or incomplete antisynthetase syndrome etc. The diagnosis may be changed, when more or new information is available.

vi). Inclusion body myositis – characterised by slowly progressive proximal and distal weakness, low CK and rimmed vacuoles on trichome stain of muscle biopsy. Not typically seen in children - few case reports only [6].

**2. Date of first symptom:** Date of first symptoms of myositis as reported by parent or patient - may include first symptoms of weakness, skin or associated features (whichever presented first).

**3. Date of diagnosis:** Date of confirmed diagnosis of myositis.

**4. Diagnostic Clinical Criteria**

**Symmetrical proximal muscle weakness:** Symmetric muscle weakness affecting proximal muscles more than distal ones and sparing eye and facial muscles, with a subacute onset (weeks to months) and with a progression. Muscle weakness should be defined by manual muscle testing or other objective strength testing.

**Heliotrope rash**: Purple, lilac-coloured or erythematous patches over the eyelids or in a periorbital distribution, often associated with periorbital oedema.

**Gottron's papules or Gottron's sign:** Gottron’s Papules: Erythematous to violaceous papules (sometimes scaly) over the extensor surfaces of joints; may occur over the MCP / PIP joints, elbows, knees, malleoli and toes. Gottron’s sign: Erythematous to violaceous macules or patches (not palpable) over the extensor surfaces of joints.

**Nailfold capillary changes:** Dilatation of periungual capillaries which may be accompanied by vessel dropout and which is visible by naked eye examination or by using additional magnification with otoscope, ophthalmoscope, or dermatoscope.

**Other characteristic JDM rash:** Characteristic rash of myositis - not due to alternative cause.

**Diagnostic Investigations**

**Muscle biopsy:** abnormal consistent with myositis (defined by histopathological report).

**Elevation of muscle enzymes -** Enzyme level above the upper range of normal according to hospital's / laboratory normal reference range (to be defined further by consensus as part of on-going work).

**EMG:** abnormal consistent with myositis.

**MRI:** abnormal consistent with myositis.

**Auto-antibody positivity –** antibody positive (above normal reference range) according to local hospital reporting (to be defined further by consensus as part of on-going work).

**C. Treatments**

If steroid or disease modifying anti-rheumatic drug or biologic given prior to diagnosis of JDM.
