# Supplementary material for: Developing a provisional, international Minimal Dataset for Juvenile Dermatomyositis: for use in clinical practice to inform research
Source: Pediatr Rheumatol Online J. 2014 Jul 21;12:31. doi: 10.1186/1546-0096-12-31 (PMC4113599; doi:10.1186/1546-0096-12-31)
Supplement: Additional file 1 — Form A: provisional definitions, Form B: provisional definitions (to be refined further as part of on-going work)[31-37] [file 1546-0096-12-31-S1.zip › add1/8866834211209620_add2.doc]

**Form B: Provisional Definitions**

For each variable mark yes (present), no (not present), not available (not available at time of entry but can be chased and added at a later date) or not done (not examined or investigated, hence not available at a later date).

**Patient Information**

**Patient ID Number:** Country specific person identification number or social security number. This field will have different use in different countries based on local regulation and legislation. You may decide to use hospital specific project numbers in this field.

**Patient name and patient birthday** are the core identifiers in the system, when you later look for the patient. If your local legislation does not allow this, details can be omitted and a reference number that is recognisable to your centre can be used. The field allows the option of entering year and month of birth without day of birth.

**Consultant:** Typically if the 'typing' user is not the consultant.

**Patient Hospital** Used when patient followed at external hospital or clinic - other than the 'typing' hospital.

**A. Growth**

**Height:** Height in cm on date of visit (recorded in centimetres with 1 figure over the decimal point range; xx.y).

**Weight:** Weight in kg on date of visit (recorded in kilograms with 1 figure over the decimal point range; xx.y).

**B. Disease activity / damage (clinical features)**

**1. Active Muscle Disease**

**Proximal muscle weakness:** Symmetric muscle weakness affecting proximal muscles more than distal ones and sparing eye and facial muscles, with a sub-acute onset (weeks to months) and with a progression. Muscle weakness should be defined by manual muscle testing or other objective strength testing.

**Other muscle weakness:** May include distal or generalised muscle weakness or bulbar muscle weakness.

**CMAS:** Childhood myositis assessment scale (CMAS) score - a validated measure of assessment of physical function in children. Maximum score = 52. Instructions can be found on the IMACS website; http://www.niehs.nih.gov/research/resources/assets/docs/cmas_instructions.pdf. There is an important discrepancy between the CMAS Scoring Sheet found on this web-site and the scoring sheet published in the original CMAS article [34] The discrepancy is due to mis-prints in that article regarding the scoring of manoeuvres 4, 8, and 12 (see Tables 2 and 3 in the 1999 article) [35]. The authors of the 1999 article acknowledge the mis-prints in that article and fully agree that the “CMAS Scoring Sheet” and the “Description of Manoeuvres” found on this web-site are correct.

**MMT8:** Enter score for each muscle group or simply add total score. MMT8 is a set of 8 designated muscles tested unilaterally (potential score 0 - 80), generally on right side (unless cannot be tested on right, then use left side). Calculation is always based on 8 scores (left side is used in calculation if right side is not set). If less than 8 muscles tested the MMT8 cannot be calculated [23].

**2. Active Skin Disease**

**Heliotrope rash:** Purple, lilac-coloured or erythematous patches over the eyelids or in a peri-orbital distribution, often associated with peri-orbital oedema.

**Gottron’s Papules:** Erythematous to violaceous papules (sometimes scaly) over the extensor surfaces of joints; may occur over the MCP / PIP joints, elbows, knees, malleoli and toes. Gottron’s sign: Erythematous to violaceous macules or patches (not palpable) over the extensor surfaces of joints.

**Nailfold capillary changes** Dilatation of periungual capillaries which may be accompanied by vessel dropout and which is visible by naked eye examination or by using additional magnification with otoscopy.

**Lipodystrophy** Loss of subcutaneous fat – may be localised, partial or generalised

**Calcinosis** Dystrophic calcium deposits, observed clinically or by imaging, which involves the skin, subcutaneous tissue, fascia or muscle.

**Cutaneous ulceration due to myositis** Extensive injury to dermis or deeper due to dermatomyositis.

**Generalised:** Widespread generalised oedema (anasarca).

**Peri-orbital** Tissue swelling around the eyes.

**Malar / facial erythema due to myositis** Confluent erythema in a malar distribution - involving the cheeks and extending over the nasal bridge (nasolabial folds often spared), or more extensive involvement of the face including periorbital, forehead, lateral face and ears.

**Shawl sign / V sign due to myositis Shawl sign**: erythema over the upper back, posterior neck and shoulders, sometimes extended to lateral arms. V sign: erythema over lower anterior neck and upper anterior chest.

**Mechanic’s hands** Scaling or cracking of the skin over the lateral or palmar aspects of the fingers or thumbs.

**Alopecia due to myositis** Hair loss - may be diffuse (non-scarring, non-erythematous widespread alopecia) or focal (patchy alopecia with scaling and erythema localized to areas of inflammation).

**Vasculopathic lesions due to myositis** Including ulceration, gingival erythema, periungual erythema / telangiectasia and digital infarction.

**Photosensitivity due to myositis** Typical skin rashes of JDM triggered by UV light.

**Livido reticularis due to myositis** Net-like / lace-like pattern of erythema.

**Other erythema due to myositis** Including erythema on the face / helix of the ear, Holster sign (erythema confined to the lateral thigh / hip), or linear extensor erythema (confluent macular erythema of skin overlying the extensor tendon sheaths).

**Panniculitis due to myositis** inflammation of the subcutaneous fat.

**3. Major organ involvement due to myositis**

**Musculoskeletal involvement due to myositis: Arthritis**: Inflammation, including swelling, warmth, tenderness, and/or redness of one or more joints detected by physical exam.

**Musculoskeletal involvement due to myositis: Contractures due to myositis:** Fixed flexion deformities in the joints due to myositis.

**Gastrointestinal involvement due to myositis: Dysphagia:** Difficulty in swallowing or objective evidence of abnormal motility of the oesophagus.

**Gastrointestinal involvement due to myositis: Dysphonia:** Change in voice due to myositis.

**Gastrointestinal involvement due to myositis: Abdominal pain or GI ulceration due to myositis (exclude peptic ulcer disease):** Ensure that other causes of abdominal pain or ulceration are excluded.

**Pulmonary involvement due to myositis – interstitial lung disease:** Radiologic (chest x-ray or chest CT scan) documentation of inflammation or scarring (fibrosis) of the parenchyma of the lung. Abnormal PFT attributable to inflammatory process or fibrosis.

**Cardiac involvement due to myositis:** Any of: Pericarditis: Inflammation of the pericardium defined clinically or by electrocardiogram (ECG) or echocardiogram; Myocarditis: Inflammation of the myocardium defined clinically or with echocardiographic or other objective evidence; Arrhythmia:  clinical or ECG evidence of irregular heart beat, Sinus tachycardia:  resting heart rate > greater than upper limit of age-appropriate normal value in a paediatric patient.

**4. Constitutional**

**Fever (>38^o^C) due to myositis:** Ensure that other causes for fever are excluded.

**Weight loss (>5%) due to myositis:** Ensure that other causes of weight loss are excluded.

**Fatigue due to myositis:** A persistent feeling of tiredness / exhaustion / need to rest because of lack of energy or strength due to myositis.

**Irritability due to myositis:** Uncharacteristically easily irritated or annoyed, not due to other causes other than myositis.

**Raynaud’s phenomenon:** Discoloration of fingertips or other acral areas (two or three colours) to emotion or cold.

**5. Other**

**Malignancy:** regardless of whether considered to be myositis associated.

**D.** **Global Disease Activity**

**1. Physician VAS – global activity (overall):** Disease activity defined as potentially reversible pathology or physiology resulting from myositis. Clinical findings known or suspected to be due to another disease should not be considered. Rate your global activity assessment judged by all the information available to you today including the patient’s appearance, history, physical examination and laboratory tests. Scored in cm with one figure after the decimal point (ie range 0.0-10.0) [23].

**2. Physician VAS – extra-muscular global activity:** Encompasses an overall evaluation for the disease activity in all the extra-muscular organ systems EXCLUDING muscle disease activity. Scored in cm with one figure after the decimal point (ie range 0.0-10.0).

**3. Parental / patient VAS:** The global evaluation by the patient, or by the parent if the patient is a minor, of the patient's overall disease activity at the time of assessment using a 10 cm visual analogue scale. Scored in cm with one figure after the decimal point (ie range 0.0-10.0).

**E. Health Questionnaire: CHAQ:** Functional questionnaire to be completed by the patient or parent. (Calculated score 0 – 3 with allowed values only ie. 0125, 0.25, 0.375 etc) [36][37]. *CHAQ may be replaced by an alternative disease-specific health questionnaire if determined appropriate after consensus.*

**F. Quality of Life (QoL) Tool:** Quality of life tool to be completed by the patient or parent *(exact QoL tool to be determined by consensus as part of on-going work)*

**G. Investigations**

**1. Blood tests- muscle enzyme elevation**

**For all enzymes**: Enzyme level above the upper range of normal according to hospital's normal reference range *(to be defined further by consensus as part of on-going work)*

**2. Abnormal investigation since last visit (consistent with flare of myositis)**

**MRI:** abnormal consistent with myositis.

**Muscle biopsy:** abnormal consistent with myositis.

**EMG:** abnormal consistent with myositis.

**Lung function tests:** abnormal due to myositis (not due to an alternative or co-existing diagnosis).

**ECG / ECHO:** abnormal due to myositis (not due to an alternative or co-existing diagnosis).

**H. Treatment**

**1. Treatment start date (mm/yyyy):** Month and year patient first received treatment for myositis.

**For all drugs:** Patient taking at time of visit (or recently taken since time of last data entry into database).

**Other treatment:** Other disease modifying treatment for myositis not included in list above (name drug name in free text box).
